# Ventralis oralis anterior (Voa) deep brain stimulation plus Gamma Knife thalamotomy in an elderly patient with essential tremor: A case report

**DOI:** 10.1097/MD.0000000000025461

**Published:** 2021-04-16

**Authors:** Byeong Ho Oh, Young Seok Park

**Affiliations:** a Department of Neuroscience, Graduate School; b Department of Neurosurgery, Chungbuk National University Hospital, College of Medicine, Chungbuk National University; c Department of Neurosurgery, Gamma Knife Icon Center, Chungbuk National University Hospital; d Institute for Stem Cell and Regenerative Medicine (ISCRM), Chungbuk National University, Cheongju, Republic of Korea.

**Keywords:** deep brain stimulation, essential tremor, Gamma Knife radiosurgery, old age, ventralis oralis anterior nucleus

## Abstract

**Rationale:**

Deep brain stimulation (DBS) of the ventralis intermedius nucleus (Vim) provides a safe and effective therapy for medically refractory essential tremor (ET). However, DBS may be risky in elderly patients and those with ischemic brain lesions. Gamma Knife radiosurgery (GKS) is a minimally invasive procedure, but bilateral thalamotomy is dangerous.

**Patient concerns:**

We report a case of ventralis oralis anterior nucleus (Voa) DBS for dominant hand tremor plus Voa GKS for nondominant hand tremor in a very elderly patient with medically intractable ET.

**Diagnosis:**

An 83-year-old right-handed woman visited our hospital with a medically intractable ET. Because of the ischemic lesion in the right basal ganglia, we decided to perform left unilateral DBS instead of bilateral DBS.

**Intervention:**

We chose Voa as the target for DBS because, clinically, her tremor was mainly confined to her hands, and Voa had better intraoperative microelectrode recording results than Vim.

**Outcomes:**

After 2 years, her right-hand tremor remained in an improved state, but she still had severe tremor in her left hand. Therefore, we performed GKS targeting the right Voa. One year after surgery, the patient's hand tremor successfully improved without any complications.

**Lessons:**

Salvage Voa GKS after unilateral Voa DBS is a valuable option for very elderly patients and patients with ischemic brain lesions. We suggest that Voa GKS thalamotomy is as useful and safe a surgical technique as Vim GKS for dystonic hand tremor. To the best of our knowledge, this is the first case report using salvage Voa as the only target for ET.

## 1 Introduction

Tremor is a hyperkinetic movement disorder characterized by rhythmic oscillations of one or more body parts. This disorder may be disabling and significantly impairs the quality of life. However, it is sometimes challenging to find the appropriate treatment to reduce disability^[1]^ as pharmacological treatment of severe tremor is often disappointing. Medications are always well tolerated and often provide only limited benefits.^[2]^ No major breakthrough in such medical treatments has recently emerged.^[3]^

Deep brain stimulation (DBS) is an increasingly popular and standard option for disabling tremor whenever it is insufficiently improved by drug treatment. However, in certain conditions, it increases surgical risk, making it difficult to perform bilateral DBS. For elderly or high-risk patients, there are options such as utilizing unilateral or staged operations or selecting thalamic targets if patients suffer tremor-dominant symptoms.^[4–6]^

Gamma Knife thalamotomy is also a safe and effective noninvasive approach to the treatment of essential tremor (ET) and is worthy of consideration in many patients. This procedure is especially relevant as a treatment option for medically refractory tremor in the elderly or in those with contraindications to DBS.^[7]^

Traditionally, the ventralis intermedius nucleus of the thalamus (Vim) has been used for a target for DBS in most tremor syndromes. This clinical practice of using Vim is largely based on excellent tremor outcomes in patients with refractory ET and Parkinson disease (PD).^[8]^

Nevertheless, despite the exceptional results observed in some patients, thalamic stimulation may not successfully treat all patients. Additionally, uncommon forms of tremors remain challenging to treat. Thalamic lesioning surgery and DBS for complex tremor syndromes have yielded mixed results and, in many cases, disappointing results.^[9,10]^

To date, there have been no reports of combined DBS of the ventralis oralis anterior nucleus (VOA) for dominant hand tremor and Gamma Knife thalamotomy of the Voa for nondominant hand tremor in very elderly tremor patients. Therefore, we introduce a surgical strategy for certain patients who are very elderly with high surgical risks or damaged ischemic brain lesions. Herein, we report Voa DBS for dominant hand tremor plus Voa Gamma Knife thalamotomy for the nondominant side in very elderly medically intractable ET with ischemic brain lesions.

## 2 Case report

An 83-year-old right-handed female patient presented with a 13-year history of tremor. Because the characteristics of her tremor were mainly postural tremor and the tremor limited her ability to eat, wash, and other activities of daily living, we determined that the patient's symptoms met the diagnostic criteria for ET.

She had undergone extensive medical therapy, but all of them provided only transient improvements in the tremor. Upon examination, she had severe intention and mixed with dystonic tremor in both hands (Table 1).

**Table 1:** Clinical rating scale for tremor (CRST).

CRST score	Baseline	At 6month after left DBS	At 6month after right GKS
Part A (tremor)	R: 50	R: 37	R: 37
	L: 50	L: 50	L: 38
Part B (task)	R: 20	R: 5	R: 5
	L: 20	L: 20	L: 6
Part C (disability)	R: 32	R: 15	R: 15
	L: 32	L: 32	L: 17
Total (a maximum of 120)	R: 102	R: 57	R: 57
	L: 102	L: 102	L: 61

**Figure 1:**
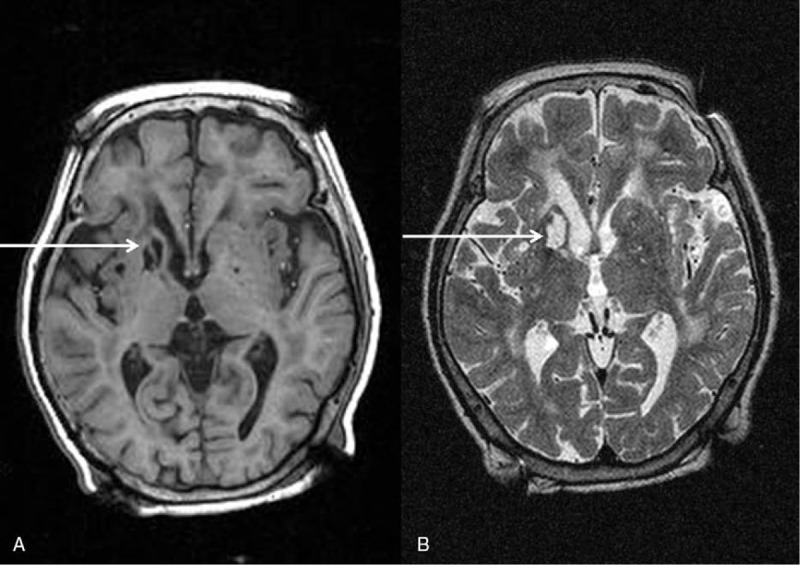
T1 (A) and T2 (B) signals from preoperative MRI showed changes associated with ischemic leukomalacia at the head of the right caudate nucleus (white arrow), which were regarded as sequalae of past cerebral ischemia. Electrode insertion into the ischemia-damaged brain carries a risk of further damage or hemorrhage.

Significantly, her tremor was mainly confined to both hands while moving. Parkinsonism was excluded by physical examination and levodopa response. On admission, her physical and mental condition was normal. No other neurological abnormalities, except tremor, were found. and other laboratory investigations were clear, but a brain magnetic resonance imaging (MRI) revealed sequelae of chronic infarction of the right basal ganglia (Fig. 1).

To decide the surgical procedure, we should consider the right basal ganglia lesion. Bilateral DBS could be risky for her brain condition, and bilateral Gamma Knife radiosurgery (GKS) is also dangerous. Therefore, we decided to treat her dominant hand tremor preferentially via unilateral DBS.

Her dystonic hand tremors were characteristically confined in both hands, and Voa was reported to be superior when mixed with dystonic tremor. We tested both Vim and Voa during intraoperative microelectrode stimulation, and Voa was observed to be superior to Vim. Therefore, we decided to perform DBS for Voa rather than Vim.

### 2.1 Surgical method-DBS

The patient underwent stereotactic implantation of a left thalamic deep brain stimulator and a left-sided implantable pulse generator under local anesthesia. The procedure included placement of a Leksell stereotactic head frame (Elekta Instruments AB, Stockholm, Sweden) and preoperative MRI targeting involving identification of the anterior (AC) and posterior commissure (PC).

The initial target was set as follows: 13 mm lateral to the AC-PC line and 7.1 mm (33.3%) anterior to the PC, at the level of the AC-PC line. quadripolar electrodes (Medtronic 3389, Minneapolis, MN) were successfully implanted into the target.

**Figure 2:**
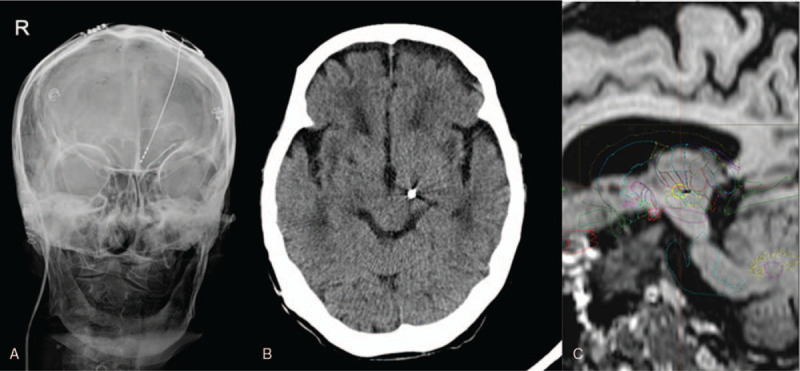
Post. DBS Skull X-ray (A) and axial CT (B) placement of the electrode in the left ventralis oralis anterior nucleus. A sagittal MRI image (C) superimposed on the Schaltenbrand–Wahren atlas using planning software.

Intraoperative stimulation with the macroelectrode completely suppressed the patient's right-hand tremor. Furthermore, the clinical response and microelectrode recording were superior in Voa than Vim. Therefore, we decided to place the electrode in Voa rather than Vim (Fig. 2AB).

After surgery, the stimulator was adjusted by the following parameters: monopolar stim (C+, 1-) 2.8 V, pulse width 90 μs.

Six months after surgery, the patient's right-hand tremor dramatically improved, and she was able to eat and write by herself. (Table 1).

Two years later, at the time she visited our hospital, full suppression of the right-hand tremor was reminded, but she still had severe tremor in her left hand. For the sake of her quality of life, she and her family were eager for any surgical procedure that could improve the function of her left hand as the previous surgery had done for her right hand.

We considered her right basal ganglia ischemic lesion and concluded that GKS may be more appropriate than DBS. Additionally, the tremor features were mainly confined to the hand, and the results of previous Voa DBS were good. We decided to perform GKS on the right Voa.

### 2.2 Surgical method–GKS

Considering surgical risk and damaged ischemic brain, staged DBS is not a good option for her status, and we suggest salvage Voa GKS for nondominant tremor. We decided to use GKS for the contralateral side because electrode insertion into the ischemic damaged brain could pose a substantial risk of further damage or hemorrhage.

The Leksell coordinate frame G was attached to the patient's head. A 1.5-tesla Philips MRI was acquired, and contrast-enhanced T1 and T2 images were taken at 1 mm. The anterior commissure, PC, and third ventricle were identified. Stereotactic radiosurgical thalamotomy was performed using the Gamma Knife Icon (Elekta AB, Stockholm, Sweden). The target was localized to the Voa nucleus of the right thalamus (Fig. 2C). Radiosurgical planning was performed using Leksell GammaPlan software (Elekta Instrument, Stockholm, Sweden) and was set as follows: the therapeutic target was placed 3.0 mm above the AC-PC line, 13.1 mm lateral to the midline, and 0.8 mm posterior to the midpoint of AC-PC.

The final optimization of the x-coordinate was safely adjusted to avoid exceeding a dose of 18 Gy in the posterior limb of the internal capsule. A maximum dose of 130 Gy was delivered with 50% and 65 Gy, with a single 4 mm collimator.

One year after Gamma Knife thalamotomy, her left-hand tremor gradually improved over time. Her right hand was slightly better than her left hand, but there was no significant difference in scores (Table 1). No adverse complications were observed during follow-up. Before surgery, she always depended on a caretaker for daily living, but now, she can use both hands for eating, washing, and dressing, and so she can care for herself independently.

## 3 Discussion

DBS has been associated with surgical complications, including wound infection, pneumonia, hemorrhage, pulmonary embolism, neurologic sequelae, and even death.^[11,12]^ Several authors recommend that unilateral or staged unilateral DBS surgery is safer than simultaneous bilateral DBS surgery.

Although bilateral electrode implantation is considered to be a “standard approach,” unilateral DBS also improves motor symptoms and quality of life, reduces the need for medication, and possibly enhances mental flexibility.^[13]^ Furthermore, unilateral surgical procedures can be less frequently associated with severe complications (intracranial, extracranial, and hardware-related) than bilateral procedures.^[14,15]^

When considering bilateral DBS, there is an ongoing debate on the DBS implantation approach: staged unilateral DBS vs simultaneous bilateral DBS.

The simultaneous bilateral approach offers patient convenience and presumed healthcare cost savings because it requires only one operative procedure for lead implantation. The presumed disadvantage of simultaneous bilateral DBS is increased pre and postoperative complication rates due to increased intraoperative time. The advantages of unilateral staged DBS include decreased continuous intraoperative time, leading to a presumed decreased risk of pre and postoperative complications. The disadvantages of staged unilateral DBS include a delay in time to therapeutic efficacy, 2 hospital stays, and 2 frame placements for the patient. Given the decreased continuous intraoperative time, many groups have elected to use unilateral staged DBS in elderly patients, especially those over the age of 70.^[5]^

DBS of the Vim has been established as a standard surgical treatment method for medically refractory tremor. The advantages of DBS are that it can be controlled after surgery and it takes a shorter time than GKS to improve tremor; however, surgery is difficult and carries greater risk in elderly patients than in the average population, and there are special situations (i.e., anticoagulant treatment, high infection risk, intracranial lesion, explanted lead) when such treatments are the best option.^[16]^ On the other hand, Gamma Knife thalamotomy does not require the creation of burr holes or a cranial electrode puncture and is a comparatively minimally invasive procedure. Therefore, it requires a shorter recovery time than DBS.

Indications for Gamma Knife thalamotomy include severe disabling tremor, contraindications for Vim DBS, and refusal to undergo DBS. There are several causes of severe tremor that are not well controlled by standard pharmacological treatments. The most common tremor treated by gamma knife thalamotomy is ET, which increases in incidence and severity with advancing age.^[17]^

Most reports have demonstrated the efficacy of unilateral GKS. Evidence of the efficacy of simultaneous or staged bilateral Gamma Knife thalamotomy remains limited. In addition, there are concerns about the side effects of bilateral thalamotomy. Thalamic lesions have several potential side effects, including dysarthria, paresis, ataxia, and gait and balance problems, because of the proximity of the Vim to the corticobulbar tract.^[18]^ Therefore, simultaneous bilateral Gamma Knife thalamotomy is not recommended for patients with bilateral tremor.

The Vim is located near the ventralis oralis posterior nucleus (Vop) (according to Hassler thalamic nomenclature).^[19]^ Whereas Vim is the cerebellar receiving area of the motor thalamus, the Voa, and Vop are pallidal receiving areas.^[19]^ An effect on rigidity therefore appears to reflect the influence of GKS on the Vop and pallidothalamic fiber tracts toward the Voa.

Voa has been considered less important than other targets of neuromodulation surgery. Recently, however, some uncommon movement disorders that are difficult to treat using existing methods have been effectively treated by Voa.

Goto et al reported that thalamotomy involving the Voa and Vop of the thalamus successfully alleviated the writer's cramp.^[20]^ Taira reported that dystonic symptoms almost completely disappeared after ventro-oral thalamotomy in 12 patients with writer's cramp.^[21]^ Horisawa et al reported that both radiofrequency thalamotomy and Gamma Knife thalamotomy targeting Voa for musician's dystonia achieved beneficial effects.^[22,23]^ Ghika et al performed bilat. Voa DBS as salvage surgery after an unsuccessful bilat. Pallidal DBS or postanoxic generalized dystonia.^[24]^ Asahi et al. successfully treated a patient with drummer's dystonia using RF thalamotomy to the left Voa.^[25]^ Foote et al. reported that complex stimulation of both Vim and Voa-Vop using dual electrodes achieved better results than Vim stimulation alone in posttraumatic Holmes tremor and multiple sclerosis tremor.^[9,26]^

Therefore, stereotactic surgery targeting Voa is considered effective in patients with focal task-specific dystonia and other uncommon movement disorders. Additionally, Voa has been suggested as a secondary addable target to overcome tolerance.^[27,28]^

In our case, bilateral DBS could be risky because the patient was very old and had ischemic brain lesions. Bilateral GKS is also associated with a risk of bilateral thalamotomy. Therefore, we performed unilateral DBS for dominant hand tremor preferentially and unilateral GKS for nondominant hand tremor subsequently. Because the intraoperative microelectrode response of Voa was superior to that of Vim during DBS surgery, we performed DBS for Voa rather than Vim. After Voa DBS, the patient's dominant hand tremor improved. Therefore, we decided to perform GKS for non-dominant tremor at the Voa rather than the Vim.

This method may not be applicable to all medically intractable ET patients who are very old and have ischemic brain lesions. The characteristics of the patient's tremor, underlying disease, brain condition, and intraoperative response should be fully considered before this method is performed. Additionally, the patient's consent and volition must come first. To evaluate the efficacy of this method, additional studies including more cases and long-term follow-up are needed.

## 4 Conclusion

Voa DBS for the dominant hand plus Voa GKS for the nondominant hand is a safe strategy in very elderly patients. Elderly patients may have brain ischemic lesions, and DBS for ischemic lesions can be dangerous in certain patients. If bilateral DBS is risky, salvage GKS for the other side is helpful for relieving symptoms and reducing surgical risk. In addition, Voa GKS thalamotomy is also a useful and safe surgical technique similar to Vim targeting in elderly patients with dystonic hand tremors. Salvage Voa GKS after unilateral Voa DBS is a salvage option for very elderly patients or those with ischemic brain damage. This strategy increases patient tremor outcomes and quality of life at a relatively low risk.

## Author contributions

**Conceptualization:** Young Seok Park.

**Supervision:** Young Seok Park.

**Writing – original draft:** Byeong Ho Oh.

**Writing – review & editing:** Young Seok Park.
